# Emotional reactions of peers influence decisions about fairness in adolescence

**DOI:** 10.3389/fnhum.2013.00745

**Published:** 2013-11-12

**Authors:** Eduard T. Klapwijk, Sabine Peters, Robert R. J. M. Vermeiren, Gert-Jan Lelieveld

**Affiliations:** ^1^Department of Child and Adolescent Psychiatry, Curium – Leiden University Medical CentreLeiden, Netherlands; ^2^Institute of Psychology, Leiden UniversityLeiden, Netherlands; ^3^Leiden Institute for Brain and Cognition, Leiden UniversityLeiden, Netherlands

**Keywords:** interpersonal effects of emotions, adolescence, social value orientation, dictator game, social interactions, development

## Abstract

During adolescence, peers take on increasing importance, while social skills are still developing. However, how emotions of peers influence social decisions during that age period is insufficiently known. We therefore examined the effects of three different emotional responses (anger, disappointment, happiness) on decisions about fairness in a sample of 156 adolescents aged 12–17 years. Participants received written emotional responses from peers in a version of the Dictator Game to a previous unfair offer. Adolescents reacted with more generous offers after disappointed reactions compared to angry and happy reactions. Furthermore, we found preliminary evidence for developmental differences over adolescence, since older adolescents differentiated more between the three emotions than younger adolescents. In addition, individual differences in social value orientation played a role in decisions after happy reactions of peers to a previous unfair offer, such that participants with a “proself” orientation made more unfair offers to happy peers than “prosocial” participants. Taken together, our findings demonstrate that adolescents take emotions of peers into account when making social decisions, while individual differences in social value orientation affect these decisions, and age seems to influence the nature of the reaction.

## INTRODUCTION

Emotions play a pivotal role in social interactions, particularly during adolescence, a life stage of significant social development (Blakemore, 2008). For instance, research in adults showed that being in a happy mood themselves makes people rely more on available cues and schemas about their interaction partner (Lount, 2010) and makes them more cooperative in negotiations (e.g., Forgas, 1998). In addition, emotions expressed by others also affect the perceiver’s behavior in (further) interactions with the expresser (Parkinson, 1996; Van Kleef et al., 2010). These interpersonal effects of others’ emotions have not yet been studied in adolescence and are therefore the focus of the current study.

Social functional accounts of emotions (e.g., Frijda, 1986; Parkinson, 1996; Keltner and Haidt, 1999; Morris and Keltner, 2000; Elfenbein, 2007; Van Kleef, 2009; Van Kleef et al., 2010) posit that one important function of emotion expression is communication to influence the behavior of others. Expressed emotions – either facial, verbal, or postural – may trigger affective reactions and inferences about the other person that influence subsequent social interactions with this person (Van Kleef, 2009).

In adults, interpersonal effects of emotions have been fruitfully investigated in negotiation settings and allocation games (e.g., Van Kleef et al., 2004; Kopelman et al., 2006; Lelieveld et al., 2012; for a review, see Van Kleef et al., 2010). Generally, these studies have demonstrated that bargainers react differently to distinct emotional expressions when responding to others. However, the effects of emotions may depend on the situational context and can be influenced by individual personality differences such as social value orientation.

### INTERPERSONAL EFFECTS OF ANGER, DISAPPOINTMENT, AND HAPPINESS

The emotions used in the current study include anger, disappointment and happiness. These three emotions were selected because they enable us to compare both the effects of negative and positive communicated emotions, as well as the effects of different types of negative emotions (anger vs. disappointment). Studying discrete emotional expressions is important since they carry more information than the more vague and diffuse (negative vs. positive) category of moods (Frijda, 1986; Van Kleef et al., 2010). Another reason to include anger, disappointment, and happiness in this study is that we wanted to know if results for adolescents would differ from the results of adults in a previous study by Lelieveld et al. (2013a).

When being confronted with anger, the perceiver’s affective reactions may cause reciprocal anger (leading to competition) or complementary fear (leading to concessions; Lelieveld et al., 2012). Furthermore, someone else’s anger can also lead to the conclusion that it might be more beneficial to concede before the situation will escalate. Indeed people make higher concessions to angry compared to happy opponents, because of the toughness communicated by the angry person (Van Kleef et al., 2004). However, communicating anger from a low power position (i.e., when having a low influence on the other’s outcomes) might backfire; in these situations the angry recipient gets offered less (Van Dijk et al., 2008; Lelieveld et al., 2013a). Although disappointment – like anger – is a negative emotion, its interpersonal effects differ from the effects of anger. The few studies on the interpersonal effects of disappointment have shown that communicated disappointment invokes feelings of guilt (Lelieveld et al., 2013b), leading to higher offers compared to communicated anger (Van Kleef et al., 2006; Lelieveld et al., 2011, 2012). Finally, happy reactions in negotiations may lead to increased liking and higher subsequent offers in cooperative settings. In more competitive settings people will infer that the happy other is satisfied, which will take away the need to concede and encourage lower offers (Van Kleef et al., 2004, 2010).

### INDIVIDUAL DIFFERENCES

The interpersonal effects of emotions may partly depend on individual personality differences. One important personality trait that has consistently been shown to influence one’s bargaining behavior (e.g., McClintock and Liebrand, 1988; Parks, 1994; Van Lange et al., 2007), and, more specifically, the impact of emotions of others on one’s bargaining position (e.g., Van Kleef and Van Lange, 2008), is a person’s social value orientation (SVO; Messick and McClintock, 1968). Social value orientation is the dispositional preference for distributions of outcomes for the benefit of either self or others. Persons with a prosocial SVO try to maximize both one’s own outcomes and the other’s outcomes and to minimize differences in outcomes for themselves and others. Proselfs are more interested in maximizing differences between themselves and others (i.e., competition) or they are solely concerned about their own outcomes with little or no interest in others’ outcomes (i.e., individualism; Van Lange et al., 1997). One previous study found that proselfs were more likely to concede than prosocials after a disappointed reaction from the other player in a multi-round negotiation, likely due to strategic motivations (Van Kleef and Van Lange, 2008). In order to look at the direct effects of the communicated emotions on prosocials and proselfs, we will investigate the interpersonal effects of emotions in a situation without strategic considerations (i.e., in the case of the Dictator Game). In such situations one might expect prosocials to be more affected by other’s emotions, because they are more empathic than proselfs (Declerck and Bogaert, 2008). To test this hypothesis, we decided to consider the effects of social value orientation in the current study.

### INTERPERSONAL EFFECTS OF EMOTIONS IN ADOLESCENCE

Research with allocation games in the domain of interpersonal effects of emotions has mainly focused on adults. To our knowledge, no previous study has focused on the interpersonal effects of emotions in adolescence. However, studying this topic during adolescence is relevant for several reasons. First, notable social changes are seen during this life stage. There is an increased focus on peer relationships and an improvement in social skills that are used to form more complex social relations (Steinberg and Morris, 2001). Second, some studies suggest that the capacity to recognize facial emotions of all six basic emotions (i.e., happiness, sadness, anger, fear, disgust, and surprise) is still developing throughout adolescence and into adulthood (e.g., McGivern et al., 2002; Herba and Phillips, 2004; Wade et al., 2006; Thomas et al., 2007). Hence, it might be harder for (younger) adolescents to differentiate between emotions. Third, adolescents increasingly take the situational context, such as the perspective of their interaction partner, into account during social interactions. Changes in fairness views continue to impact sharing in allocation games throughout adolescence (Crone, 2013). For example, when allowed to distribute jointly earned resources, older adolescents share more with peers that earned more (Almås et al., 2010). Older adolescents also make more use of knowledge about the intentions of others when offering money or when evaluating offers made by others (Güroğlu el al., 2009). Moreover, an increase in social cognitive abilities with adolescence has also been shown in other complex perspective-taking tasks (Dumontheil et al., 2010) and the capacity to attribute mental states to others (i.e., theory of mind) has been found to improve during and beyond adolescence. For example, older adolescents are faster at attributing emotional mental states to others than younger adolescents (Keulers et al., 2010) and adolescents make more errors than adults when evaluating complex theory of mind stories (Vetter et al., 2012). In order to bring these findings together, we studied how adolescents use emotional information from peers during allocation games. This can be an important novel approach to characterize the effects of emotions of peers during social interactions in adolescence.

### THE CURRENT STUDY

In the current study, we therefore investigated interpersonal effects of emotions on allocations in adolescence. We used a procedure developed by Lelieveld et al. (2013a), in which we examined participants’ choices in a Dictator Game after receiving verbal emotional reactions from a peer (depicting disappointment, anger, or happiness) to a previous unfair offer. In the Dictator Game (Kahneman et al., 1986), one player divides an amount of money between oneself and another player. The other player is forced to accept this – the dictator’s – offer. The Dictator Game allows one to study the interpersonal effects of emotions in a clear and controlled setting. Allocators do not need to consider whether a low offer will be rejected (as opposed to the Ultimatum Game, where the other player can reject the offer), which minimizes the interference of strategic motivations.

This study will test the following hypotheses. First, in line with the results from Lelieveld et al. (2013a), we hypothesized that angry reactions from peers to a previous unfair offer would lead to more unfair offers compared to receiving happy statements in response to identical unfair offers (Van Dijk et al., 2008; Lelieveld et al., 2013a). In addition, we expected less unfair offers in reaction to disappointed compared to angry reactions because disappointment leads to a concern for the outcomes of others (Lelieveld et al., 2011, 2012, 2013a). Second, we explored age differences in the amount of unfair offers for the three distinct emotions. Given the increasing incorporation of the situational context with age (Güroğlu et al., 2009; Almås, et al., 2010; Dumontheil et al., 2010) and adolescents’ heightened susceptibility to peer influence (Gardner and Steinberg, 2005), we explored if older adolescents would differentiate more between the three emotions than younger adolescents. Third, we investigated effects of individual differences in SVO (i.e., prosocials vs. proselfs). Previous research has shown that the effects of disappointment depend on a person’s SVO (Van Kleef and Van Lange, 2008), which we extend by examining the effects of SVO on anger, disappointment as well as happiness. We expected participants with a proself orientation to make more unfair offers compared to participants with a prosocial orientation and to differentiate less between the emotional expressions of others (cf. Van Lange et al., 1997).

## MATERIALS AND METHODS

### PARTICIPANTS

The final sample of participants included 156 adolescents (76 girls) aged 12.41–17.75 years (*M* = 15.17, SD = 1.22) who were recruited from local secondary schools in The Netherlands. All had then experienced a social change from primary to secondary school. Participants were recruited from schools whose populations have common Dutch ethnicity. Self-report indicated that 97.4% of the participants was born in The Netherlands and that 79.6% had two parents with a Dutch background; 22.4% had at least one parent that was born in another country. No information about socioeconomic status and (history of) psychiatric disorders was collected. In total, 209 eligible participants initially took part in the study, of which 19 were excluded because they did not choose an unfair option in the first phase of the experiment (see below). Of the remaining 190 participants, there was incomplete data for 28 participants due to computer problems, who were therefore excluded from further analyses. A 10-min time limited version of the Standard Raven’s Progressive Matrices (Raven et al., 1998) was administered to assess fluid reasoning skills in order to obtain an estimate of general intellectual ability. Since we used a time limited version of the Raven, raw test scores were used. Participants who scored more than 2 SD from the mean on this measure were excluded from further analysis (*N* = 6), because we wanted our participants to be of comparable intelligence. Informed consent was obtained from all participants and from primary caregivers.

### EXPERIMENTAL TASK

Using a procedure developed by Lelieveld et al. (2013a), we examined participants’ choices in a Dictator Game after receiving emotional reactions to a previous unfair offer from others.

#### Phase one

In the first phase of the experiment, participants read a scenario presented on paper in which they negotiated for a company. They were instructed to divide 10 tokens between themselves and another person. Participants chose between two predetermined distributions of the 10 tokens in two different scenarios. First, they had to choose between a 6–4 distribution in favor of themselves and a 5–5 equal distribution. Second, participants chose between a 6–4 distribution in favor of themselves and a 4–6 distribution in favor of the other. The negotiation scenario was meant to create a business setting, in order to assure that most participants chose the 6–4 option in this phase of the study. We wrote in the scenario that we are researchers interested in monetary negotiations by companies that try to maximize their profits, and that we are interested in how youths think about these negotiations. Adolescents were instructed to imagine which option they would choose if they were part of a company and had to make a profit.

Indeed, most participants (190 participants out of 209) chose a 6–4 distribution in at least one of the two scenarios. To ensure credibility of the second phase, only these 190 participants took part in phase two of the experiment.

#### Phase two

The second phase took place 1 week after the first phase, to support the credibility of the researchers collecting reactions from others to the offer. In this phase, participants were told that their unfair offer (the 6–4 distribution) was presented to 60 same-aged peers who were given the opportunity to write out their reaction upon receiving the offer. In reality, the 60 reactions were pre-programmed and rated for emotional significance in a pilot-study with adult participants (see Lelieveld et al., 2013a). On each trial, participants read one of the reactions to the unfair offer, presented on a computer screen. Subsequently, they played a version of the Dictator Game with the person who provided the emotional reaction, in which participants were again allocators in a Dictator Game and divided 10 tokens. Participants could now choose between a 7–3 distribution (i.e., 7 tokens for themselves and 3 for the other) and a 5–5 distribution. We did not include a 6–4 distribution, to ensure that a desire to be consistent with the participants’ first offer did not influence the results. We emphasized that the (simulated) recipients did not know that their written reactions would be sent back to the participant to ensure that participants trusted the emotional reactions to be non-strategic (i.e., not aimed to influence the participant’s new offer). There was no business setting in this phase anymore; participants now played as individuals. They were told that they would play for real money and that after the experiment, three participants from every class would be selected by a computer to get some rounds actually paid out. Since participants were playing for real money (at least some of their rounds could get paid out), we have no reason to assume participants did not believe the cover story. Additionally, participants in the Lelieveld et al. (2013a) did not express any doubts about the cover story either.

On each trial, participants were paired with a different player, whose first name (indicating gender) was provided. Emotional reactions were either angry, disappointed, or happy. Examples of emotional reactions depicting anger were “I feel really angry after receiving this offer,” “This annoying person really pisses me off,” and “I am starting to get really furious right now.” Examples of reactions depicting disappointment were “This really disappoints me,” “I expected more from the other person,” and “I am really disappointed in the other person.” Examples of reactions depicting happiness were “I am really happy with this offer,” “The other person made my day,” and “This is perfect, I am really satisfied” (see also Lelieveld et al., 2013a).

### QUESTIONNAIRES

#### Social value orientation

We measured social value orientation (Messick and McClintock, 1968) with a nine-item questionnaire developed by Van Lange et al. (1997). Participants can choose from three different options to allocate valuable points between themselves and a recipient. Responses can be categorized in accordance with three different social value orientations: “prosocials,” who maximize outcomes for themselves and the recipient and minimize differences between these outcomes; “individualists,” who maximize their own outcomes without regard for the recipient’s outcomes; and “competitors” who maximize their own outcomes relative to the recipient’s outcomes. Participants were classified into one of these categories when they made six or more consistent choices. Participants who did not meet this requirement were excluded from further analyses that investigated relations with SVO. Similar to several previous studies (e.g., De Cremer and Van Vugt, 1999; Olekalns and Smith, 1999; Joireman and Duell, 2005; Van Dijk and De Cremer, 2006; Van Kleef and Van Lange, 2008), we chose to combine individualists and competitors into one category, the “proselfs,” in order to compare self-interest and collective interest. In our sample, 35% of the participants were classified as prosocials, 50% as proself, and 15% could not be classified.

## RESULTS

For phase two of the experimental task, several participants informed us that the experiment was too long and that toward the end of the task, it became difficult to still concentrate on the emotional reactions. To ensure that we were analyzing meaningful results, we decided before any analyses were performed to limit our analyses to the first 30 trials. We made this decision based on a trade-off between statistical power and motivation of participants. By analyzing only half the trials, we ensured in the best possible way that participants were sufficiently motivated for all trials while still retaining statistical power. Our analyses on these trials showed that when collapsing all types of emotional reactions together, participants chose an unfair 7–3 distribution in a mean of 49% of the trials (SD = 19%).

To check for differences in unfair choices between the three emotional reactions, we performed a repeated-measures analysis of variance (ANOVA) with emotion (anger vs. disappointment vs. happiness) as a within-subjects variable and percentage of unfair choices as the dependent variable. This analysis yielded a main effect of emotion, *F*(2,310) = 4.58, *p* = 0.02, η^2^ = 0.03. Least significant difference (LSD) *post hoc* tests showed that participants chose the unfair option more often when dealing with angry recipients (*M* = 51%, SD = 33%, *p* < 0.001) or happy recipients (*M* = 53%, SD = 31%, *p* = 0.01) than when dealing with disappointed recipients (*M* = 43%, SD = 31%). In other words, disappointed reactions of a peer to a previous unfair offer led to more generous offers than angry or happy reactions. There was no difference in the amount of unfair offers for angry and happy recipients (*p* = 0.53). Although we did not expect any sex differences we explored an effect of sex, which was not found, *F*(2,308) = 0.52, *p* = 0.52, η^2^ = 0.003.

To investigate the time course of the responses for the different emotions, we compared the percentage of unfair offers per emotion for the first trial and the last trial (i.e., the 10th trial for each emotion). A repeated-measures ANOVA with emotion and trial number as between-subjects variables indicated that there was a slight interaction between emotion and trial number, *F*(2,310) = 3.06, *p* = 0.051. This indicates that there was a marginally significant effect of trial number, i.e., that there were differences in the percentage of unfair offers per emotion over the course of the task. Because this effect is quite small and we averaged over 10 responses per emotion, we expect that the effect of trial number is relatively small.

### AGE EFFECTS

**FIGURE 1:**
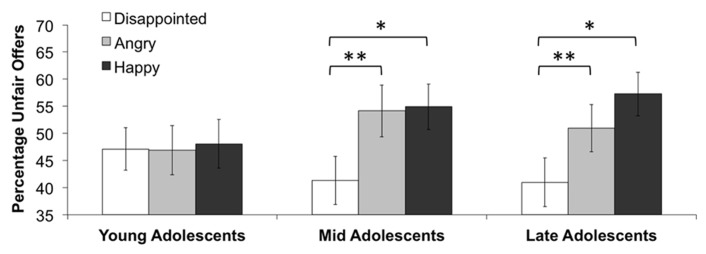
**Percentage of unfair offers for the three emotions, separate for three age groups.** Single asterisk (*) indicates *p *< 0.05; double asterisk (**) indicates *p *< 0.01.

Collapsed over all emotions, no correlation was found between the total amount of unfair distributions and age (*r* = 0.10, *p* = 0.21). We also checked for effects of age for the three emotions separately, by performing a repeated-measures ANOVA with emotion (anger vs. disappointment vs. happiness) as a within-subjects variable and percentage of unfair choices as the dependent variable, with age as a covariate. No effects of age were found, *F*(2,308) = 1.34, *p* = 0.26, η^2^ = 0.01. We also divided our sample in three similar sized age groups: young adolescents (*M* = 13.75 years, SD = 0.65 years), mid adolescents (*M* = 15.29 years, SD = 0.32 years), and late adolescents (*M* = 16.49 years, SD = 0.40 years). See **Table 1** for details about the age groups. There was no significant difference between the age groups for sex, Χ^2^ = 2.26, *p* = 0.32, SVO, Χ^2^ = 0.07, *p* = 0.97, and Raven scores, *F*(2,132) = 1.01, *p* = 0.37, η^2^ = 0.02. No significant interaction was found for the three emotions and age group, *F*(4,306) = 1.11, *p* = 0.34, η^2^ = 0.01 (see **Figure 1**). However, based on our expectations that younger adolescents would differentiate less between the different emotions than older adolescents, we looked at the interpersonal effects of emotions for each age group separately. We conducted a repeated-measures ANOVA with emotion (anger vs. disappointment vs. happiness) as a within-subjects variable and percentage of unfair choices as the dependent variable separately for each age group. We found no main effect of emotion in young adolescents, *F*(2,102) = 0.02, *p* = 0.92, η^2^ < 0.001, but we did find a trend for mid adolescents, *F*(2,104) = 3.22, *p* = 0.06, η^2^ = 0.06, and a significant effect for late adolescents, *F*(2,100) = 3.57, *p* = 0.05, η^2^ = 0.07. LSD *post hoc* tests indicated that for young adolescents none of the effects of the emotions differed from each other, that for mid adolescents, disappointment differed from anger (*p* = 0.001) and happiness (*p* = 0.04), and that for late adolescents, disappointment also differed from anger (*p* = 0.005) and happiness (*p* = 0.03). The effects of anger and happiness did not differ from each other in any age group (all *p*s > 0.05). **Figure 1** depicts the means for each age group, and shows that with increasing age, adolescents seem the differentiate more between the three emotions. That is, the emotions seem to affect the adolescents differently with increasing age.

**Table 1:** Demographic characteristics of the three age groups.

Age group	Young adolescents	Mid adolescents	Late adolescents
*N*	52	53	51
Age range	12.41–14.69	14.78–15.89	15.92–17.75
Mean age (SD)	13.75 (0.65)	15.29 (0.33)	16.49 (0.40)
*N* females	22	25	29
*N* proself SVO	26	26	26
*N* prosocial SVO	17	19	18
Mean % unfair (SD)	47.37 (18.29)	50.13 (20.58)	49.74 (17.28)
Mean raven (SD)	34.06 (6.13)	35.62 (5.50)	35.57 (6.43)

### EFFECTS OF SOCIAL VALUE ORIENTATION

**FIGURE 2:**
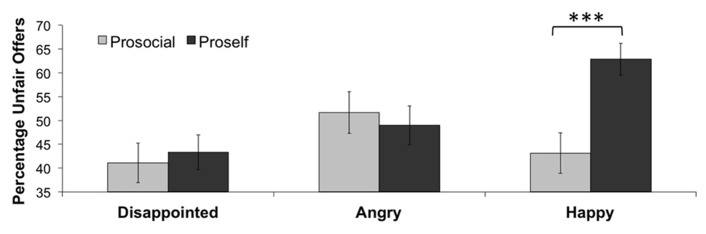
**Percentage of unfair offers for the three emotions, separate for prosocial and proself SVO.** Triple asterisk (***) indicates *p *< 0.001.

Collapsed over all emotions, prosocials made less unfair offers than proselfs, although this effect is slightly above significance level, *t*(130) = 1.92, *p* = 0.057, *d* = 0.34. Furthermore, a repeated-measures ANOVA with emotion as a within-subjects variable and social value orientation (proself vs. prosocial) as a between-subjects variable yielded a significant interaction effect, *F*(2,260) = 4.37, *p* = 0.03, η^2^ = 0.03 (see **Figure 2**). Follow-up *t*-test comparisons indicated that proselfs proposed more unfair offers than prosocials, but only after happy emotional reactions, *t*(130) = 3.68, *p* < 0.001, *d* = 0.67. We found no age effects for this analysis, that is, no further interactions of SVO and age were found. We also performed these analyses separate for the three age groups. We only found an interaction between emotion and SVO in the late adolescent group, *F*(2,84) = 4.61, *p* = 0.03, η^2^ = 0.10. Follow up *t*-test comparisons showed that this effect is again due to proselfs proposing more unfair offers than prosocials, but only after happy reactions, *t*(42) = 3.04, *p* = 0.004, *d* = 0.9.

## DISCUSSION

In this study we investigated adolescents’ choices in a Dictator Game after receiving angry, disappointed, and happy emotional reactions from others to an unfair offer. We found that adolescents took information about emotional reactions into account when they were allocating resources. Even though the participant had a lower payoff for the fair than for the unfair option, disappointed reactions led to more generous offers than angry or happy reactions. Happy reactions were more likely to evoke a tendency to act in a self-interested manner, especially amongst participants with a “proself” social value orientation. Young adolescents seemed to differentiate less between the three emotions compared to late adolescents.

### ADOLESCENTS VS. ADULTS

Based on previous research with adult participants (Van Dijk et al., 2008; Van Kleef et al., 2010; Lelieveld et al., 2013a), we hypothesized that anger would lead to more unfair offers than happiness and disappointment. Angry expressions indeed led to more unfair offers than disappointed expressions, which emphasizes the importance of studying discrete emotional expressions instead of valence alone. Both anger and disappointment are reactions to undesirable behavior of others, and both communicate a wish for a behavioral change in the other (Van Dijk and Zeelenberg, 2002). However, a difference between disappointment and anger is that disappointment might lead to more fair offers via a feeling of guilt, whereas anger might instead lead to more unfair offers when communicated from a low-power position (which was the case in our study) because of reciprocal anger (Lelieveld et al., 2011, 2012). Disappointment also differs from anger and happiness because it is regarded more complex than the basic emotions of anger and happiness. However, research suggests that disappointment and anger are two of the most frequently expressed negative emotions (Van Dijk and Zeelenberg, 2002; Lelieveld et al., 2011). We were interested how different negative emotions that signal dissatisfaction would impact offers by adolescents. Future studies may include other basic emotions such as sadness to contrast different basic emotions.

The current results from an adolescent sample differ partly from previous findings using the same paradigm with adults (Lelieveld et al., 2013a). The adolescents in our study made more unfair offers in response to happy and angry reactions compared to disappointed reactions, while the adults in the Lelieveld et al. (2013a) study only made lower offers in response to angry (and not happy) reactions. When reading happy reactions the adolescents possibly inferred that the happy other was satisfied with the previous unfair offer, and accordingly chose more unfair offers (Van Kleef et al., 2010). This is in line with the idea that the general function of positive emotions is to serve as a cue to continue the current course of action (Cacioppo and Gardner, 1999). Previous studies with adults showed this pattern of utilization of happiness as well (Van Kleef et al., 2004). However, the effect of happiness was mainly driven by the proself (SVO) participants in the oldest age group in our sample (see below).

### AGE EFFECTS

Our analyses did not reveal any effects of age on the total amount of unfair offers. Previous studies also found that fairness preferences (as measured with the Dictator Game) do not seem to change substantially after childhood (Gummerum et al., 2008; Güroğlu et al., 2009; Steinbeis et al., 2012). However, since older adolescents are better at considering consequences of their decisions for others (Crone, 2013) and we expected the adolescents to increasingly take the situational context (i.e., different emotional reactions) into account when growing older, we analyzed effects of emotional reactions separately for three age groups. Although we did not find an interaction between age and the offers after the different emotions, we did find differences when we compared the interpersonal effects of the discrete emotions within the separate age groups. This analysis suggested that the youngest adolescents did not differentiate between the three emotions, while older adolescents did. Only the older adolescents made less unfair offers in response to disappointed expressions compared to angry and happy expressions.

Although future studies are needed to confirm these results, the finding that younger adolescents seem to make less use of information conveyed in others’ emotions than older adolescents is in line with previous research on emotion recognition and perspective-taking. Possibly, younger adolescents are less able at distinguishing the written emotional expressions, as previously observed for facial expressions of the six basic emotions (i.e., happiness, sadness, anger, fear, disgust, and surprise; Wade et al., 2006) and anger and fear specifically (Thomas et al., 2007). However, we have no reason to believe that adolescents did not perceive the correct emotion from the different reactions, since most disappointed, angry, and happy statements contained the words disappointed, angry, or happy respectively (or any clear synonym). Verbally presented emotions might also be processed differently with increasing age, as was found in younger children (Jenkins and Ball, 2000). In addition, more mixed feelings in response to social emotion scenarios have been linked to pubertal development in young adolescents (Burnett et al., 2011b), suggesting more complex understanding of verbally described emotions with advancing puberty. Hence, the difference between the youngest and oldest adolescents in the current study seems to reflect an improvement with age in understanding emotions and incorporating emotional information into decision-making.

In addition to behavioral evidence, several neuroimaging studies have revealed that brain regions important for social decisions are changing during adolescence (reviewed in Burnett et al., 2011a; Crone and Dahl, 2012). For example, age-related increases in temporo-parietal junction (TPJ) activation were observed when adolescents played the Trust Game, in which it is important to take the perspective of the other player into account (Van den Bos et al., 2010). In adults, heightened TPJ activation in response to happy reactions was found using the paradigm employed in the current study (Lelieveld et al., 2013a). An interesting but speculative direction for future research would be to investigate if the increased amount of unfair offers in response to happiness is related to lower TPJ activity in adolescents compared to adults.

### SOCIAL VALUE ORIENTATION

In order to evaluate individual personality differences in responses to others’ emotions we measured social value orientation. The hypothesis that proselfs would make more unfair offers in general than prosocials was confirmed, although the effect was only marginally significant. Additionally, as considered above, the higher amount of unfair offers in response to happy reactions in our study can be attributed to proself (SVO) participants who made more unfair offers than prosocial participants in this condition (see **Figure 2**). It is possible that proselfs perceived the other’s happiness with a previous unfair offer as a signal of satisfaction that could be answered with more unfairness. Prosocials may have liked the happy others more and therefore may have made less unfair offers. Previous studies have demonstrated that proselfs tend to be more concerned about their own outcomes in allocation games than prosocials (Parks, 1994; Balliet et al., 2009). In the current study, proselfs tended to be somewhat more selfish in general, but not univocally egocentric. Proselfs did not differ from prosocials in their choices after disappointed and angry expressions. It appears that proselfs only act more selfishly in certain situations (Ketelaar and Au, 2003; Van Kleef and Van Lange, 2008), such as acting upon the perceived satisfaction that was signaled by the happy others in our study. Future research could investigate these underlying mechanisms, and further explore whether SVO also modulates other positive emotions such as gratitude and hope.

### LIMITATIONS AND FUTURE DIRECTIONS

There are several limitations in the current study. First, the small effect of age that we found within our sample should be treated with caution. Our findings require replication, preferably in studies that directly compare children, adolescents, and adults. Second, the emotions in our study were verbal expressions presented on a computer screen, which may lead to concerns about ecological validity. We used verbal expressions since there is no typical facial expression for disappointment. Since emotions can be expressed in a variety of other (non-verbal) ways, future research is needed to confirm whether these effects are generalizable to more natural settings. Previous research suggests that findings with verbally expressed emotions (Van Kleef et al., 2004) are comparable with those of studies using face-to-face negotiations (Kopelman et al., 2006; Sinaceur and Tiedens, 2006) or facial emotional expressions (Pietroni et al., 2008). Furthermore, computer-mediated communication is well-established among adolescents, since the vast majority are Internet and social network users (Madden et al., 2013). However, these online interactions naturally differ from the experimental paradigm that participants engaged in for this study. Future research could investigate if similar results are found with face-to-face interactions for this particular paradigm. Third, participants reported to lose interest in the experiment toward the end of the task. The 60 trials in which participants had to choose between the same distributions may have been too repetitive. Future studies could avoid this problem by alternating between different options for fair and unfair distributions or by simply presenting less than 60 trials (e.g., 30 trials as in the current analyses). In the current study, we chose to discard the second half of the trials before performing any analyses, to avoid the inclusion of trials that are confounded by lower participant motivation and/or concentration. Finally, with the current design, we were not able to study the underlying mechanisms of participants’ fairness decisions. Future research may investigate interpersonal effects of emotions in adolescence focused on the underlying (affective) reactions; this could be realized by adding a post-questionnaire to assess feelings of, for example, guilt and anger of participants.

So far, only two studies (with adult participants) have used neuroimaging to elucidate the neural mechanisms underlying the effects of emotions in interactions (Ruz and Tudela, 2011; Lelieveld et al., 2013a). Future studies might use similar experiments to shed light on the developing brain in social interactions. Furthermore, it would be interesting to see whether psychiatric populations known for problems in empathy and emotion recognition might process interpersonal emotions in a different way, such as autism spectrum disorders or psychopathy (Blair, 2005; Kennedy and Adolphs, 2012). In addition, future work may include other discrete emotions such as sadness in order to find out whether there are other emotions that will influence younger adolescents’ fairness decisions differently. Furthermore, studies can be designed to examine the effects of emotions in situations beyond allocation games such as cooperative settings.

## CONCLUSION

The current study aimed to elucidate the role of interpersonal emotions in the context of social interaction in adolescence. In general, adolescents reacted with more generous offers after disappointed reactions from peers compared to angry or happy reactions. We also provide preliminary evidence for a developmental increase of incorporating emotional information in social decisions within adolescence. Furthermore, individual differences in SVO were related to reactions upon happiness: proselfs made more unfair offers to happy others than prosocials. Our results emphasize the importance of distinguishing between different types of emotions during social interactions in adolescence and the role of social value orientation. We hope that this study provides a fruitful starting point for the investigation of the interpersonal effects of emotions in adolescence.

## Conflict of Interest Statement

The authors declare that the research was conducted in the absence of any commercial or financial relationships that could be construed as a potential conflict of interest.
